# Co-occurrence of depression, anxiety, and perinatal posttraumatic stress in postpartum persons

**DOI:** 10.1186/s12884-023-05555-z

**Published:** 2023-04-05

**Authors:** Shelby Howard, Caitlin Witt, Karla Martin, Ateshi Bhatt, Emily Venable, Sarah Buzhardt, Andrew G. Chapple, Elizabeth F. Sutton

**Affiliations:** 1 Louisiana State University Health Sciences Center, New Orleans, LA 70112 USA; 2 Woman’s Hospital, 100 Woman’s Way, Baton Rouge, LA 70817 USA

**Keywords:** Perinatal mental health, Depression, Anxiety, Pregnancy, Perinatal posttraumatic stress

## Abstract

**Background:**

The study aim was to describe the incidence of depression, anxiety, perinatal-post-traumatic stress disorder (PTSD), and their co-occurrences in the early postpartum period in a low-resource OB/GYN clinic serving majority Medicaid-eligible persons. We hypothesized that postpartum persons screening positive for depression will have an increased risk of a positive screen for anxiety and perinatal PTSD.

**Methods:**

A retrospective study of postpartum persons receiving care in Baton Rouge, Louisiana was conducted using responses abstracted from the electronic medical record (EMR) of the Patient Health Questionnaire-9 (PHQ9), Generalized Anxiety Disorder-7 (GAD7), and Perinatal Post Traumatic Stress Disorder Questionnaire-II (PPQII). Categorical distributions were compared using Fisher exact tests, while t-tests were used to compare continuous covariates. Multivariable logistic regression was used to predict anxiety (GAD7) and perinatal PTSD (PPQII) scores while adjusting for potential confounders, as well as to predict continuous PPQII and GAD7 based on continuous PHQ9 scores.

**Results:**

There were 613 birthing persons 4–12 weeks postpartum that completed mental health screening (PHQ9, GAD7, and PPQII) between November 2020 and June 2022 as part of routine postpartum care in the clinic. The incidence of screening positive for symptoms of depression (PHQ9 > 4) was 25.4% (*n* = 156), while the incidence of positive screening for symptoms of anxiety (GAD7 > 4) and perinatal PTSD (PPQII $\ge$ 19) were 23.0% (*n* = 141) and 5.1% (*n* = 31) respectively. Postpartum patients with mild anxiety or more (i.e. GAD7 > 4) had 26 times higher odds of screening positive for symptoms of depression (PHQ9 > 4) (adjusted odds ratio [aOR] 26.3; 95% confidence interval [CI] 15.29–46.92; *p* < 0.001). Postpartum persons with a PPQII score indicating symptoms of perinatal PTSD (PPQII $\ge$ 19) had 44 times higher odds of screening positive for symptoms of depression (PHQ > 4) (aOR 44.14; 95%CI 5.07–5856.17; *p* < 0.001).

**Conclusions:**

Depression, anxiety, and perinatal PTSD are each independent risk factors for each other. To comply with the American College of Obstetricians and Gynecologists (ACOG) recommendations, providers should universally screen postpartum persons with validated screening tools for mood disturbances. However, if a complete full mood assessment is not feasible, this study provides evidence to support screening patients for depression, and if the patient screens positive, prompt additional screening for anxiety and perinatal PTSD.

## Background

Anxiety and depression are two of the most common co-morbid conditions observed in mental health disorders. Studies have reported that among persons with anxiety, 81% meet criteria for experiencing at least one lifetime episode of major depression; with risk-factors of co-morbid anxiety and depression: female gender, younger age (25–34 years), lower education level, living alone, unemployment, parental psychiatric history, and childhood trauma [1, 2].

Majority of reports on perinatal mental health focus on the occurrence of anxiety, depression, or post-traumatic stress disorder (PTSD) as discrete entities [3, 4]. However, limited studies have evaluated the occurrence of one perinatal psychiatric condition as a risk factor to predispose to another. Within the last decade, the prevalence and course of perinatal anxiety, depression, and PTSD have begun to be elucidated. An Australian study assessing symptoms of birth trauma and postnatal depression among 400 birthing persons reported 10.5% of women experienced significant distress related to childbirth and several symptoms of PTSD [3]. Another study evaluating the persistence of depression and post-traumatic stress symptoms reported symptoms did not decline over the six month postpartum period. Results showed depressive symptoms were reported in 22% of women at six weeks postpartum and 21.3% at six months postpartum and PTSD symptoms increased from 6% to 14.9% over the same time period [4].

Considering the high incidence of mood and anxiety disorders in the postpartum period, the American College of Obstetricians and Gynecologists (ACOG) recommends “all obstetric care providers complete a full assessment of mood and emotional well-being (including screening for postpartum depression and anxiety with a validated instrument) during the comprehensive postpartum visit for each patient” [5]. However, a literature review evaluating the reported use of validated screening tools by obstetricians, pediatricians, and family medicine physicians found most physician groups endorse low use of screening instruments [6, 7]. One study included in this review, reported only one in four physicians responded yes to routinely using a screening tool for depression [7].

The aim of this study was to describe the incidence of depression, anxiety, perinatal post-traumatic stress, and their co-occurrences in the postpartum period in a low-resource OB/GYN clinic where postpartum persons are universally screened for postpartum mental health disorders. The overall goal was to supplying evidence to highlight the importance of postpartum mental health screenings in clinical practice. We hypothesized that postpartum persons screening positive for depression symptoms will have an increased risk for anxiety and perinatal PTSD. To test our hypothesis, we conducted a retrospective cohort study using responses from the Patient Health Questionnaire-9 (PHQ9), Generalized Anxiety Disorder-7 (GAD7), and Perinatal Post Traumatic Stress Disorder Questionnaire-II (PPQII) matched with demographic information and birth history.

## Methods

### Study design

A retrospective study was conducted using electronic medical record data for postpartum persons completing a postpartum visit (between 4–12 weeks after delivery) in a low-resource OB/GYN clinic in Baton Rouge, Louisiana between November 2020 and June 2022. The timeframe of 4–12 weeks postpartum was chosen to capture postpartum follow-up visits but maintain consistency for reporting considering these instruments may be administered at other perinatal visits as well. 623 persons had postpartum mental health screening assessments completed over the study period between 4–12 weeks postpartum. 10 patients were excluded from the study due to missing data (*n* = 10 missing race), leaving 613 for analysis. Survey responses were universally collected as part of standard clinical practice and responses entered in the REDCap database by the research team. Demographic data was abstracted from the Woman’s Hospital electronic medical record using Structured Query Language (SQL) coding, and an oversample validated by manual chart review. The study was approved by Woman’s Hospital Foundation Institutional Review Board, with waivers of informed consent and HIPAA authorization granted.

### Outcomes

The primary outcomes were scores on mental health screening tools Patient Health Questionnaire-9 (PHQ9), Generalized Anxiety Disorder-7 (GAD7), and Perinatal Post Traumatic Stress Disorder Questionnaire-II (PPQII). The PHQ9 is a nine item screening tool with total score ranges from 0 to 27. Scores within0-4 are considered no depressive symptoms, 5–9 mild depressive symptoms, 10–14 moderate depressive symptoms, 15–19 moderately severe depressive symptoms and 20–27 severe depressive symptoms [8]. The GAD7 is a seven item screening tool for general anxiety with total score ranges from 0 to 21. Scores within0–4 are considered minimal anxiety, 5–9 mild anxiety, 10–14 moderate anxiety and 15–21 severe anxiety [9]. The PPQII assesses post-traumatic stress symptoms related to the birth experience. It is a Likert type score, with total points ranging from 0–56. Scores of 19 or higher are noted to need psychotherapy for perinatal PTSD symptoms [10].

### Statistical analysis

R statistical software version 4.0.1 was used for analyses. Categorical variables are summarized in PHQ9 scoring groups by reporting the count (%) of each category, while continuous variables are summarized by mean (standard deviation). Categorical distributions are compared across groups using Fisher exact tests, while t-tests are used to compare continuous covariates. We performed a simulated power calculation to detect an association between anxiety and depression using a two-sample test of proportions. We assumed that 20.7% of the prospective patients will have anxiety [11] and that rates of depression in patients with and without anxiety would be 81% and 22%, respectively [1, 2]. Based on this, the simulation study showed that we would have 100% power with a sample size of *N* = 600.

Multivariable logistic regression was used to predict PPQII ≥ 19 (PTSD) and GAD7 > 4 (anxiety) based on PHQ9 > 4 (depression), while adjusting for potential confounders including the alternative outcome measure. Patients with missing education status were classified as having at least a high school education in the multivariable regression. Firth’s correction was used due to separation caused by the PPQII ≥ 19 and PHQ9 > 4 relationship (all patients with PPQII ≥ 19 had a PHQ9 > 4) [12]. Lastly, multivariable linear regression was performed to predict continuous PPQII and GAD7 based on continuous PHQ9 scores. Similar analyses were performed for predicting GAD7 > 4 and PPQII ≥ 19 via logistic regression. The average treatment effects of each PHQ9 continuous score was calculated by averaging predicted continuous values and probabilities over the patient population.

## Results

### Study population and incidence of postpartum depression, anxiety, and perinatal posttraumatic stress

There were 613 unique birthing persons with available demographic information and complete mental health screening data (PHQ9, GAD7, and PPQII) between 4–12 weeks postpartum (Table 1). Among the population, 30.3% (*n* = 186) screened positive for symptoms on at least one instrument. The incidence of screening positive for symptoms of depression (PHQ9 > 4) was 25.4% (*n* = 156), and those persons were more likely to be employed (*p* = 0.012), not married (*p* = 0.01), Non-Hispanic (*p* < 0.001), and African American race (*p* < 0.001) compared to persons without depression symptoms (PHQ9 $\le$ 4). The incidence of positive screening for symptoms of anxiety (GAD7 > 4) and perinatal PTSD (PPQII $\ge$ 19) were 23.0% (*n* = 141) and 5.1% (*n* = 31) respectively (Table 1). The incidence of depression symptoms was significantly higher for patients with positive perinatal PTSD screening (100.0% vs 21.5%, *p*-value < 0.001) and for patients with positive anxiety screening (78.7% vs 9.5%, *p*-value < 0.001).

**Table 1:** Population demographics

	**All** **(*n* = 613)**	**Positive Depression Screening,** **PHQ9 > 4** **(*n* = 156)**	**Negative Depression Screening,** **PHQ9** $\le$ **4** **(*n* = 457)**	***P*-values**	**PHQ9 > 4**
Age, mean ± sd	27.0 ± 6.4	26.1 ± 6.2	27.4 ± 6.5	0.009	
Gestational age at delivery, weeks, meant ± sd	37.9 ± 2.2	37.9 ± 2.4	38.0 ± 2.2	0.883	
**Employment, n (%)**				0.012	
Unemployed	423 (69.0%)	95 (60.9%)	328 (71.8%)		22.5
Employed	190 (31.0%)	61 (39.1%)	129 (28.2%)		32.1
**Insurance, n (%)**				0.728	
Medicare/Medicaid	566 (92.3%)	143 (91.7%)	423 (92.6%)		25.3
Private/Other	47 (7.7%)	13 (8.3%)	34 (7.4%)		27.7
**Education, n (%)**				0.912	
< HS Education	137 (22.3)	34 (21.8)	103 (22.5)		24.8
HS Degree	233 (38.0)	60 (38.5)	173 (37.9)		25.8
> HS Degree	157 (25.6)	44 (28.2)	113 (24.7)		28
Missing Education	86 (14.0)	18 (11.5)	68 (14.9)		20.9
**Marital status, n (%)**				0.01	
Married	136 (22.2%)	23 (14.7%)	113 (24.7%)		16.9
Single	477 (77.8%)	133 (85.3%)	344 (75.3%)		27.9
**Ethnicity, n (%)**				< 0.001	
Non-Hispanic Ethnicity	460 (75.0%)	142 (91.0%)	318 (69.6%)		30.9
Hispanic Ethnicity	153 (25.0%)	14 (9.0%)	139 (30.4%)		9.2
**Race, n (%)**				< 0.001	
AA Race	313 (51.1%)	102 (65.4%)	211 (46.2%)		32.6
Non-AA Race	300 (48.9%)	54 (34.6%)	246 (53.8%)		18
**Postpartum Mental Health**					
**Perinatal PTSD, n (%)**					
PPQII ≥ 19 (positive screening)	31 (5.1%)	31 (19.9%)	0 (0.0%)	< 0.001	100.0
PPQII < 19	582 (94.9%)	125 (80.1%)	457 (100.0%)		21.5
**General Anxiety, n (%)**					
GAD7 > 4 (mild anxiety or more)	141 (23%)	111 (71.2%)	30 (6.6%)		78.7
GAD7 $\le$ 4	472 (77%)	45 (28.8%)	427 (93.4%)	< 0.001	9.5

### Risk factors for screening positive for anxiety in the early postpartum period (GAD7 > 4)

**Fig. 1:**
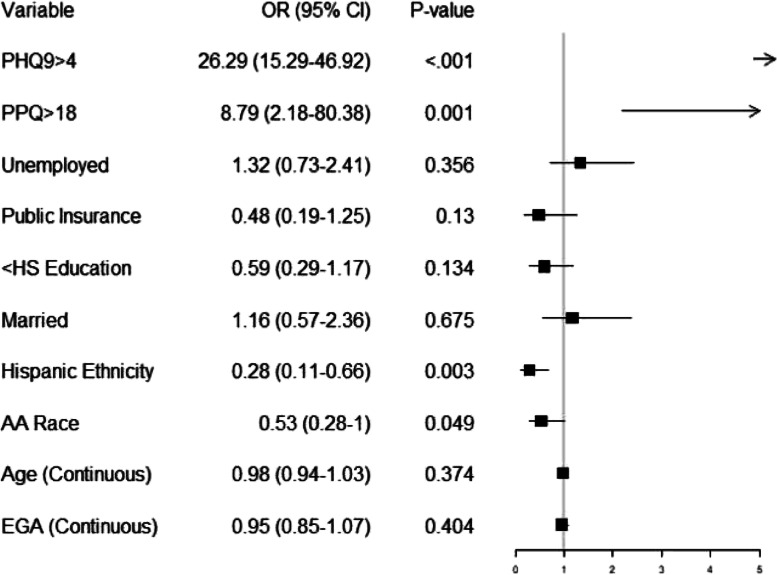
Logistic regression to predict a GAD7 score above 4

Postpartum persons with a GAD7 score indicating mild anxiety or more (i.e. GAD7 > 4) had 26 times higher odds of screening positive for symptoms of depression (PHQ9 > 4) compared to persons with a minimal anxiety score (GAD7 $\le$ 4) (adjusted odds ratio [aOR] 26.3, 95% confidence interval [CI] 15.29–46.92, *p* < 0.001; Fig. 1). The second most predictive variable of screening positive for anxiety was a positive perinatal PTSD screening. Persons who screened positive for perinatal PTSD had 8 times higher odds of also screening positive for anxiety (aOR 8.79, 95% CI 2.18–80.38, *p* = 0.001; Fig. 1). Hispanic ethnicity was also associated with 70% lower odds of screening positive for depression (aOR 0.28, 95% CI 0.11–0.66, *p* = 0.003 Fig. 1). No additional covariates evaluated (race, ethnicity, maternal age, education, insurance, or marital status) showed an effect on odds for anxiety in the early postpartum period.

### Risk factors for screening positive for perinatal PTSD (PPQII $\ge$ 19)

**Fig. 2:**
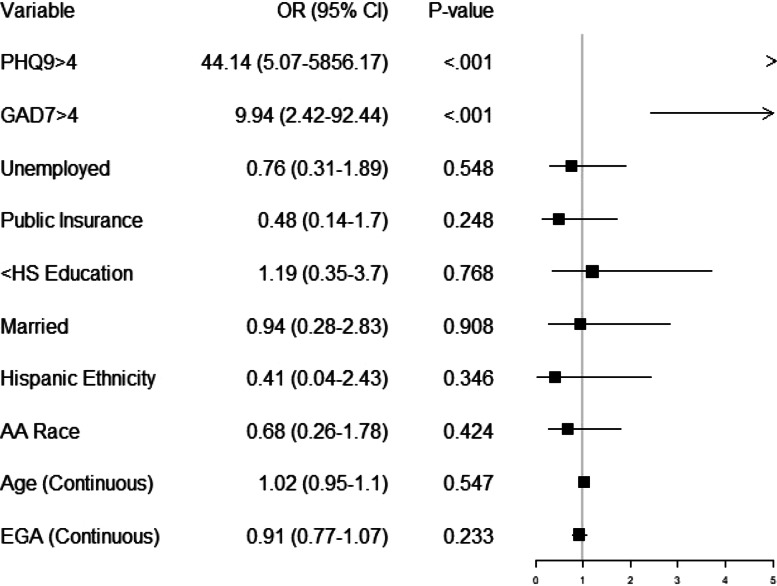
Logistic regression to predict a PPQII score 19 or greater

Postpartum persons with a PPQII score indicating symptoms of perinatal PTSD (PPQII $\ge$ 19) had 44 times higher odds of screening positive for symptoms of depression (PHQ > 4) compared to persons with a negative PPQII screening (aOR 44.14, 95% CI 5.07–5856.17, *p* < 0.001; Fig. 2). The second, and only other, predictive variable of screening positive for perinatal PTSD was a positive anxiety screening. Persons who screened positive for perinatal anxiety had 10 times higher odds of also screening positive for perinatal PTSD (aOR 9.94, 95% CI 2.42–92.44, *p* < 0.001; Fig. 2.) No additional covariates evaluated (race, ethnicity, maternal age, education, insurance, or marital status) showed an effect on odds for perinatal PTSD.

### Continuous examination of the relationship between increased continuous PHQ9 and GAD7/PPQII

**Fig. 3:**
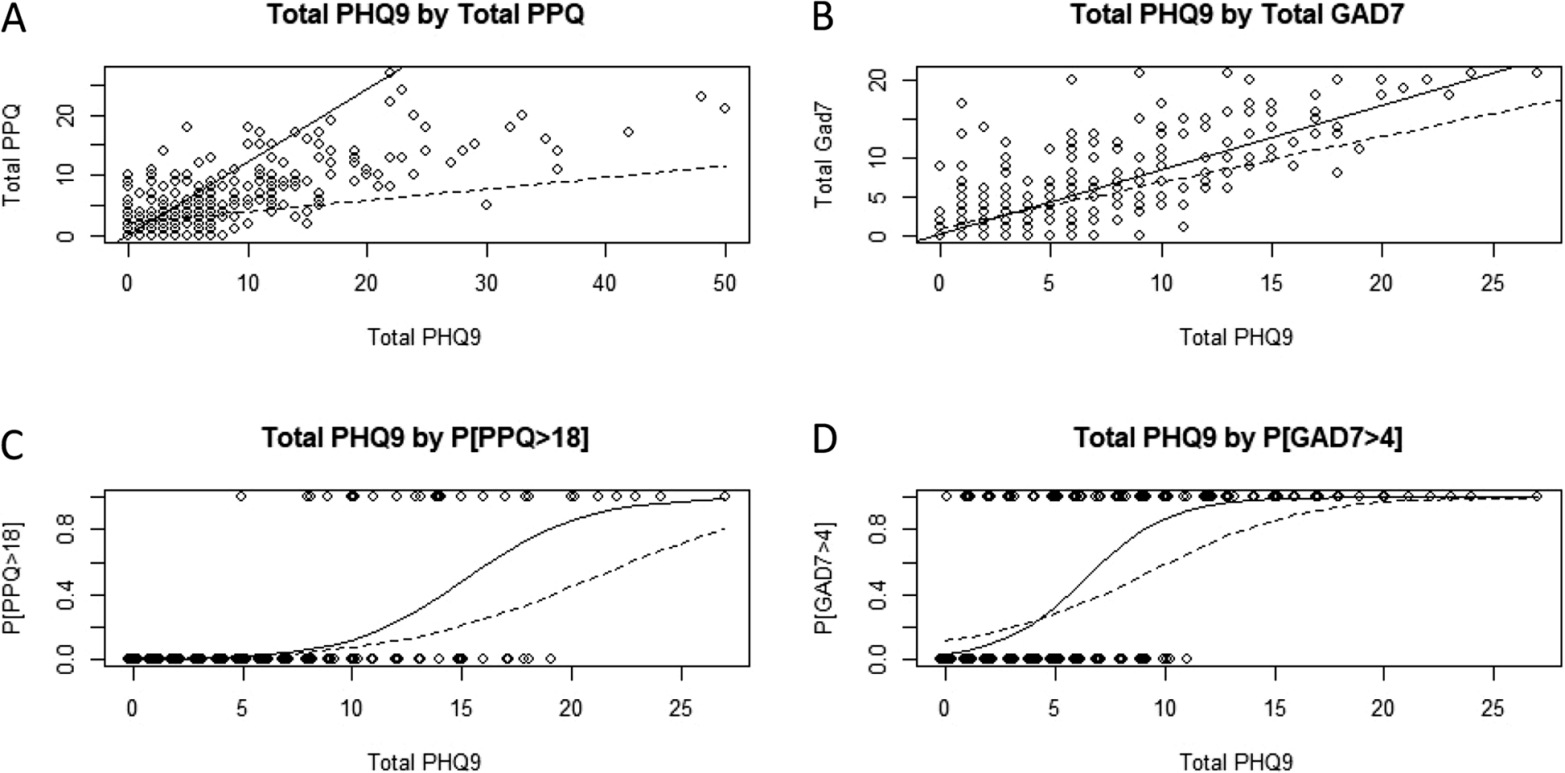
A continuous look at PHQ and GAD7/PPQ. The top plots (**A**, **B**) show relationships between continuous PHQ and GAD7/PPQ, while the bottom plots (**C**, **D**) show the relationship between continuous PHQ and GAD7 > 5 / PPQ > 18. Unadjusted linear and Firth’s corrected logistic regression are shown via solid lines, while dotted lines represent adjusted regressions for all demographic factors. These predicted values were averaged over the patient population

Lastly, we aimed to characterize the effect of continuous PHQ9 score on continuous GAD7 and PPQII scores. Additionally, we examined the relationship between PHQ9 and the probability of GAD7 > 4 and PPQII $\ge$ 19 for clinical relevance. Figure 3a and b illustrate the linear relationship between total PHQ9 and PPQII or GAD7, respectively. The dotted line represents the adjusted linear regression line for other patient covariates, while the solid line is the simple linear regression line. The adjusted lines are computed by averaging the set of patient covariates. All 4 lines showed a significant association between PHQ9 and GAD7, and PHQ9 and PPQII. Figure 3c and d display the estimated probability of GAD7 > 4 and PPQII $\ge$ 19, respectively, for unadjusted comparisons (solid line) and adjusted comparisons (dotted line). Both relationships showed a significant association, with adjusted probability estimates for PHQ9 $\ge$ 20 of $\ge$ 0.40 for PPQII $\ge$ 19 and 1.00 for GAD7 > 4.

## Discussion

Our findings demonstrate that postpartum persons are at a high risk for mood disturbances during the postpartum period. Of the study population, nearly one-third had a positive screening with at least one perinatal mental health screening instrument, mostly commonly depression. Our findings illustrate by several analytic approaches that depression, anxiety, and perinatal PTSD are intimately linked and frequently co-occur.

Our findings highlight both the prevalence and co-occurrence of symptoms of depression, anxiety, and PTSD in the postpartum period. The strongest independent risk factor for screening positive for depression was screening positive for anxiety or PTSD. In this study, scores of postpartum depression increased as scores of postpartum anxiety and PTSD increased in a linear relationship. These findings are consistent with existing literature in that symptoms of anxiety are often present in postpartum depression, with two out of three women with postpartum depression also experiencing an anxiety disorder [13]. Our work is also consistent with previous evidence for persistence of postpartum depression and PTSD symptoms co-occurring in 15% of postpartum persons at 6 months [13].

This study provides substantial clinical meaning considering the ACOG recommendations for universal postpartum mental health screening alongside evidence for the lack of said screening by clinicians [7, 8]. In past studies, only one in four providers report using validated tools when screening postpartum persons for mental health disorders. Maternal mental health should be considered a public health priority, particularly considering maternal suicide has a higher incidence of maternal mortality than hemorrhage or hypertension [14]. ACOG recommends providers complete a full assessment of mood and emotional well-being in the postpartum period [5]. Our findings suggest that a full mood assessment should include validated screening tools for depression and anxiety. A positive screen on a postpartum assessment for depression, anxiety or PTSD should prompt further evaluation with completion of assessment for all three conditions.

Our study has strengths and limitations. Strengths of this study include the universal screening approach used in clinic for screening postpartum mental health disorders. All postpartum persons were screened with validated tools for depression, anxiety, and PTSD respectively. Another strength of this study is the racial diversity included in the study population with half being African American. Our results should be interpreted with consideration of study limitations. Limitations of this study include that the PHQ9 and GAD7 are not postpartum specific screening tools and that they were developed for the general population. Further limitations are the limited diversity of socioeconomic status of the postpartum persons included in this study with 92% persons using Medicare or Medicaid insurance. Another limitation is patients comfort with expressing their concerns with their provider or filling out the survey truthfully. Some of the questions included in the PHQ9 can be common symptoms for a new parent: feeling tired, appetite changes, trouble sleeping. One subject that remains to be explored is if universal screening for postpartum depression, anxiety, and PTSD impacts postpartum and fetal outcomes. Future studies evaluating increased use of validated screening tools and the implications on treatment of mental health conditions, as well implications on health of postpartum persons and neonates.

## Conclusions

It can be concluded from this study that postpartum symptoms of depression, anxiety and PTSD are all independent risk factors for each other. The linear relationship of postpartum depression, anxiety and PTSD illustrates as scores for depression increase in the postpartum period scores of anxiety and PTSD also increase. It is important that providers caring for the postpartum population are aware of the occurrence and co-occurrence of mental health disorders in the postpartum period, due to the potential health consequences to both the postpartum person and neonate. Providers should implement full mood assessments in postpartum visits using validated screening tools for postpartum depression and anxiety as recommended by the ACOG and supported by the findings in our study. However, if unable to complete a full mood assessment, practitioners should screen patients for depression, and if the patient screens positive it should prompt the practitioner to screen for anxiety and postpartum PTSD.

## Data Availability

The datasets generated for the current study are available from the corresponding author on reasonable request.

## Abbreviations

ACOG: American College of Obstetrics and Gynecology
GAD 7: Generalized Anxiety Disorder 7
PHQ 9: Patient Health Questionnaire-9
PTSD: Post-traumatic stress disorder
PPQ II: Post Traumatic Stress Disorder Questionnaire-II
SQL: Structured Query Language
